# Magnetic resonance imaging features of alveolar soft part sarcoma: report of 14 cases

**DOI:** 10.1186/1477-7819-12-36

**Published:** 2014-02-11

**Authors:** Xubin Li, Zhaoxiang Ye

**Affiliations:** 1 Department of Radiology, Tianjin Medical University Cancer Institute and Hospital, National Clinical Research Center for Cancer, Key Laboratory of Cancer Prevention and Therapy, Tianjin; Huanhuxi Road, Hexi District, Tianjin 300060, China

**Keywords:** Alveolar soft part sarcoma, Magnetic resonance imaging

## Abstract

**Background:**

The purpose of this study was to assess the magnetic resonance imaging findings of alveolar soft part sarcoma.

**Methods:**

Magnetic resonance images of pathologically proven alveolar soft part sarcoma in 14 patients were retrospectively reviewed, including lesion location, size and shape, border definition, signals on T1-weighted and T2-weighted images, presence or absence of peritumoral and intratumoral flow voids, and enhancement pattern.

**Results:**

Patients included five women and nine men, ranging in age from 27 to 54 years, with a mean age of 36 years. A slow-growing mass without pain was the chief complaint. Eight patients had pulmonary metastases at presentation. Ten lesions arose from the extremities, two were located in the gluteal regions, one affected the presacral space and one occurred in the back. The mean maximal size of the lesions was 9.8 cm, ranging from 6.2 to 16 cm. All lesions appeared as a round (*n* = 2), ovoid (*n* = 8) or irregular (*n* = 4) shape with ill-defined margins. The lesions mainly demonstrated isointense or mildly hyperintense compared to muscle on T1-weighted images, and heterogeneous high signal intensity on T2-weighted images. Peritumoral edemas were observed in six patients. Ten lesions showed intense inhomogeneous enhancement after contrast. Intra- and peritumoral tubular flow voids representing tortuous dilated vessels with rapid blood flow were present in all cases.

**Conclusions:**

Alveolar soft part sarcoma has some distinctive magnetic resonance imaging features including a slow-growing, large mass in the soft tissue of the extremities in young adults, with numerous signal voids on T1-weighted and T2-weighted images, and strong enhancement after contrast.

## Background

The 2002 World Health Organization classification of soft-tissue tumors included alveolar soft part sarcoma (ASPS) in the category 'Tumors of Uncertain Differentiation’ [1]. ASPS is a rare malignant soft tissue tumor affecting mainly adolescents and young adults. It can present in any region of the body, but is most commonly seen in the deep soft tissues of the extremities. ASPS usually acts as a relatively indolent but relentless sarcoma, characterized by late metastases and an extended clinical course [2]. However, when radically resected, local recurrence is rare and long-term survival is possible [3]. Thus, preoperative distinction of ASPS from other soft tissue tumors may be important for optimal patient management and therapeutic planning to improve the prognosis.

Diagnostic imaging plays a crucial role in not only lesion identification but also preliminary lesion characterization. Magnetic resonance imaging (MRI) is the favored imaging modality for evaluation of this lesion because of its excellent soft tissue contrast, multiplanar imaging capability and lack of radiation exposure. Except for case reports, there have been few studies on MRI findings of ASPS [4-6]. The purpose of this study was to assess the MRI features of ASPS that contribute to its differential diagnosis from other, more common, soft tissue tumors in daily practice.

## Methods

The present study was approved by the institutional review board of Tianjin Medical University Cancer Institute and Hospital. The requirement for informed consent was waived for this retrospective study. Fourteen patients with ASPS proved by pathology in our hospital between July 2007 and June 2013 were enrolled in this study.

Of the 14 patients undergoing MRI, 10 patients underwent post-contrast T1-weighted imaging (T1WI). All MRI examinations were performed on a 1.5 T MRI system (Signa excite 1.5 T, GE Healthcare, Milwaukee, WI, USA). Fast spin-echo pulse sequences were used in these patients. Un-enhanced MRI consisted of axial and coronal T1WI, axial and coronal T2-weighted imaging (T2WI), and axial and coronal fat-suppressed T2WI. Post-contrast T1WI with or without fat-suppressed technique were acquired in the axial, coronal and sagittal planes. The imaging parameters were: for T1WI, repetition time 440 to 800 ms, echo time 8 to 12 ms; for T2WI, repetition time 2,900 to 3,020 ms, echo time 80 to 90 ms; excitations, two to three; matrix, 256 × 192 or 320 × 192; field of view, 16 × 16 cm or 34 × 34 cm; section thickness, 4 mm; and interval gap, 1.0 mm.

Clinical histories were reviewed, including patient age, sex and clinical presentation. Two experienced musculoskeletal radiologists reviewed the MR images in consensus. The analysis of MR images included the location, shape (spherical, ovoid or irregular), margin (well defined, partially ill defined or wholly ill defined), size in centimeters (largest diameter), signal intensity, presence or absence of peritumoral and intratumoral flow voids, and presence and pattern of contrast material enhancement. Skeletal muscle was used as the reference tissue for signal intensities on T1WI and T2WI. The pattern of enhancement was graded subjectively but in consensus as homogeneous enhancement, heterogeneous enhancement and peripheral enhancement.

## Results

These patients consisted of five women and nine men, ranging in age from 27 to 54 years, with a mean age of 36 years. All patients presented with a chief complaint of a slow-growing mass without pain. The duration of symptoms before referral to our hospital ranged from one week to two years. Eight patients had pulmonary metastases at presentation.

**Figure 1:**
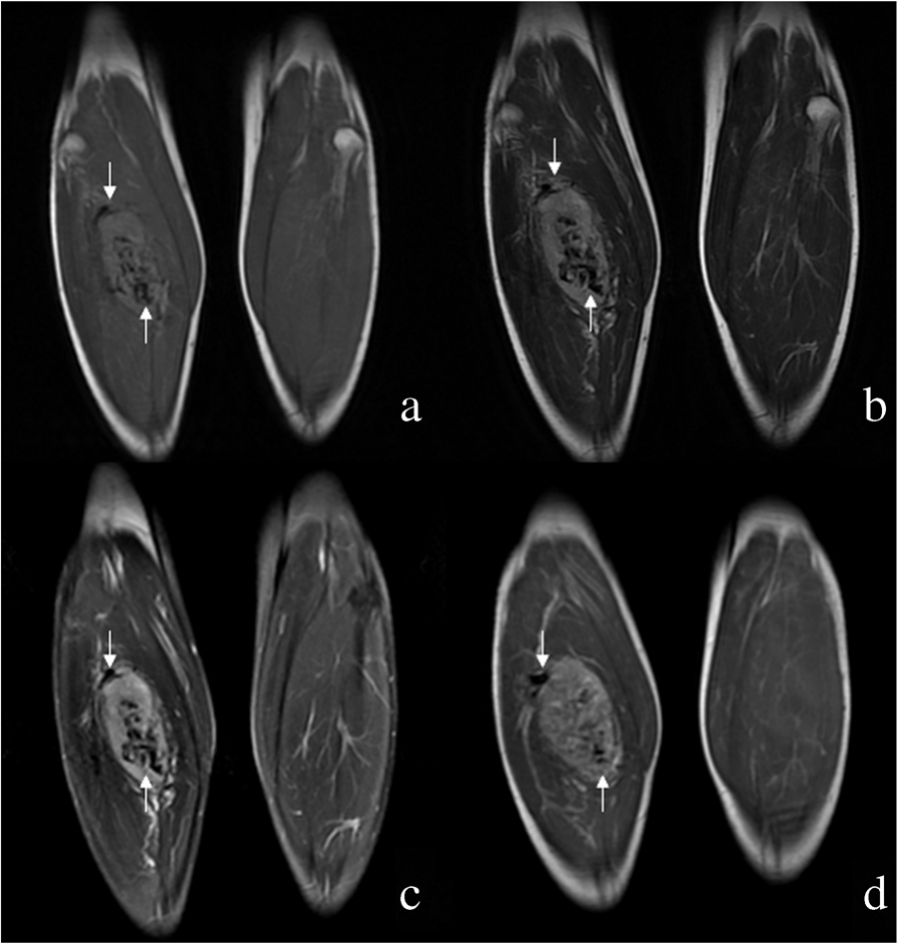
**A 25-year-old man with alveolar soft part sarcoma. (a)** Coronal T1-weighted image shows a hyperintense signal tumor in the right lower calf with an ill-defined margin in a 25-year-old man. There are tortuous intra- and extratumoral signal voids (arrows). **(b)** Coronal T2-weighted image and **(c)** coronal fat-suppressed T2-weighted image reveal the hyperintense signal tumor with intra- and extratumoral signal voids (arrows). **(d)** Coronal post-contrast T1-weighted image displays inhomogeneous enhancement of the tumor with tortuous vessel (arrows).

**Figure 2:**
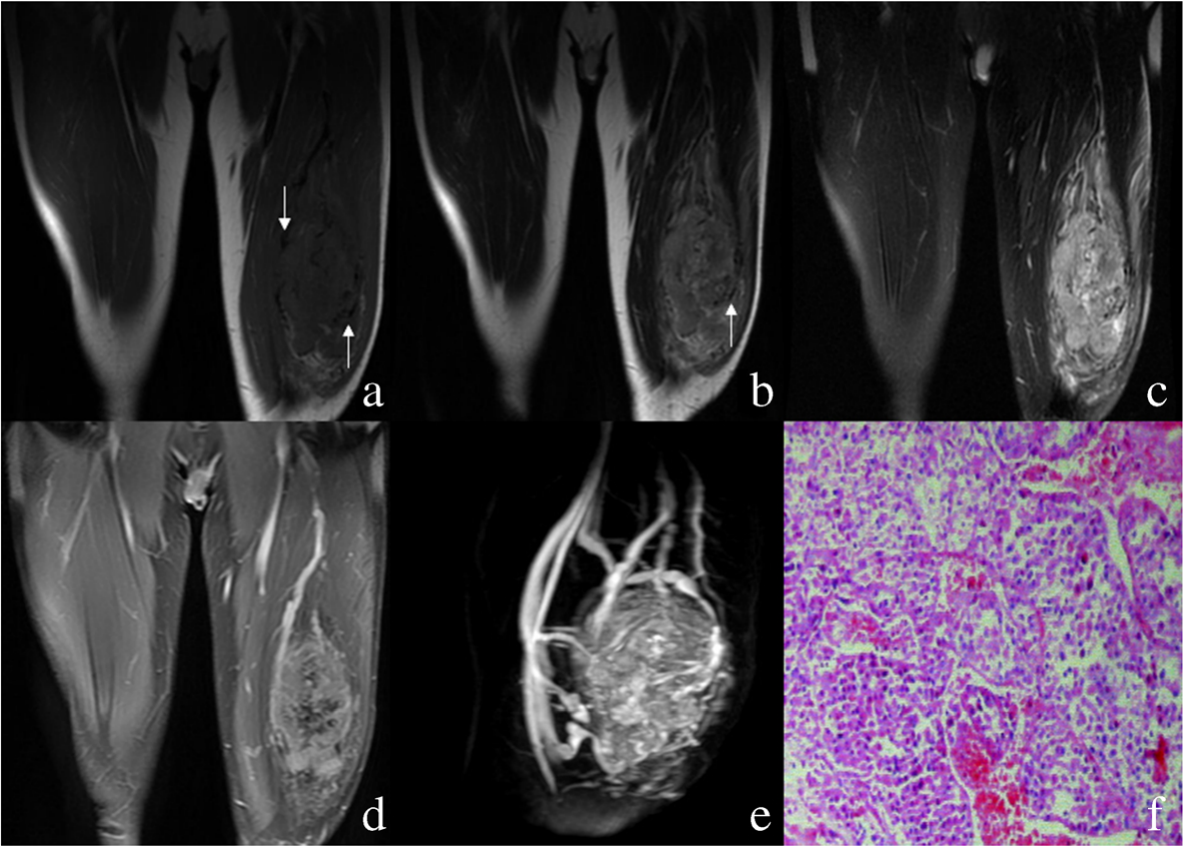
**A 27-year-old man with alveolar soft part sarcoma. (a)** Coronal T1-weighted image shows an isointense signal tumor in the left thigh muscle with an ill-defined border in a 27-year-old man. There are intra- and extratumoral tubular flow voids (arrows). **(b)** Coronal T2-weighted image reveals the hyperintense signal tumor with signal voids (arrows). **(c)** Coronal fat-suppressed T2-weighted image displays the hyperintense signal tumor with peritumoral edema. **(d)** Coronal fat-suppressed post-contrast T1-weighted image demonstrates intense inhomogeneous enhancement of the tumor. **(e)** Magnetic resonance angiography obtained from dynamic gadolinium-enhanced imaging study shows tortuous, dilated vessels in and around the tumor. **(f)** A photomicrograph shows oval to polygonal cells with eosinophilic cytoplasm and a nest-like pattern of arrangement (Hematoxylin and eosin, ×20).

ASPS was most commonly located in the extremities (*n* = 10), followed by gluteal region (*n* = 2), presacral space (*n* = 1) and the back (*n* = 1). The mean maximal size of the lesions was 9.8 cm, ranging from 6.2 to 16 cm. All lesions appeared as a round (*n* = 2), ovoid (*n* = 8) or irregular (*n* = 4) shape with partly or wholly ill-defined borders. MRI revealed that the lesions mainly demonstrated isointense or mildly hyperintense compared to muscle on T1WI, and heterogeneous higher signal intensity compared to muscle on T2WI. Peritumoral edema on fat-suppressed T2-weighted sequence was seen in six patients. Intra- and peritumoral tubular areas of flow void on both T1WI and T2WI, representing rapid blood flow in distended vessels, were present in all cases (Figures 1 and 2). Of the lesions, ten masses after contrast MRI demonstrated intense inhomogeneous enhancement (Figures 1 and 2).

Histological examinations of the excised or biopsy specimens were undertaken on all patients and demonstrated an alveolar pattern of tumor cells with uniform nuclei, abundant eosinophilic cytoplasm, and prominent nucleoli separated by thin fibrovascular stroma.

## Discussion

ASPS is an extremely rare malignant tumor that accounts for less than 1% of all soft tissue sarcomas [7]. It is caused by a specific unbalanced translocation, der(17)t(X:17)(p11;p25), that results in the formation of an ASPL-TFE3 fusion gene [2]. This tumor affects mainly adolescents and young adults. There is a slight female preponderance in patients aged ≤30 years, in contrast to a male preponderance among patients aged *>*30 years [8]. The most common site of origin is the lower limb, followed by the trunk and upper extremity, whereas in infants and children, most cases occur in the head and neck, often in the orbit or tongue. Although it behaves as a relatively indolent sarcoma, representing a slow-growing painless mass, ASPS has a high propensity for metastasis. The lungs are the most frequent site of metastasis by the hematogenous route, followed by brain and bone. In our study, in agreement with the literature, most tumors occurred in young adults. The mean patient age at diagnosis was 36 years, ranging from 27 to 54 years. The primary tumors were most commonly located in the extremity (*n* = 10), representing a relatively indolent mass, and averaged 9.8 cm (range, 6.2 to 16 cm) in largest diameter, indicating late recognition. Moreover, in our series, 10 patients had metastases at the time of initial diagnosis, all with pulmonary locations. These features are consistent with the previously reported literature, except for a slight male preponderance (*n* = 9).

ASPS is a markedly hypervascular lesion, a feature reflected on imaging studies. Regarding the radiological findings, the tumors in our series mainly demonstrated equal or slight hyperintensity compared to muscle on T1WI and heterogeneous high signal intensity on T2WI. The exact cause of the high signal intensity of ASPS on T1WI has not yet been elucidated. Some investigators think it can be attributed to slow-flowing blood in or around the tumor [4,9].

As previously reported, ASPS is usually a well-circumscribed mass [5,10]. However, in our study, all lesions had poorly defined margins and peritumoral edema was also observed in six cases. A larger tumor size (9.8 cm, the mean largest diameter) in our series, indirectly reflecting a longer disease history and tumor biological aggressiveness, might explain the difference.

Multiple intra and extratumoral signal voids on MRI are frequently observed in ASPS [4,5]. In the review of our cases, such serpentine flow voids at the core and at the margins of the tumors on all pulse sequences were observed in all cases. The signal voids were attributed by previous authors to tortuous dilated vessels with rapid blood flow, which were documented by histological findings. Of the lesions, 10 masses following contrast administration of gadolinium demonstrated strong inhomogeneous enhancement due to the presence of abundant blood vessels. Our MRI findings were compatible with ASPS, which was finally confirmed by pathology.

On the basis of MRI findings, the lesion might mimic arteriovenous malformations due to the tortuous vessels, especially on magnetic resonance angiography. In ASPS, there are significant soft tissue components surrounded by vascular tissues. By contrast, arteriovenous malformations comprise pure vascular tissue without any accompanying tissues. Another differential diagnosis is hypervascular metastasis, in which case the previous malignancy history of the patient should be sought [11].

This study was limited by its retrospective analytic nature and bias in that observers were not blinded to the diagnosis. Another limitation was the small number of cases owing to the rarity of the tumor. Moreover, angiography was not performed in any of the cases to reflect the hypervascular features of the tumor. Despite these limitations, we believe our results add to a marked understanding of the MRI appearance of ASPS.

## Conclusions

ASPS should be considered a possible diagnosis when a slow-growing, large mass with high signal intensity on both T1WI and T2WI, multiple peritumoral and intratumoral tortuous signal voids, and intense enhancement on contrast-enhanced MRI are seen in the soft tissue of the extremities, particularly in a young adult patient. Recognition of these characteristic MRI findings may lead to the early diagnosis of ASPS, which is important in the treatment of a small primary tumor.

## Consent

Written informed consent was obtained from the patients for publication of this paper and any accompanying images. A copy of the written consent is available for review by the Editor-in-Chief of this journal.

## Abbreviations

ASPS: alveolar soft part sarcoma; MRI: magnetic resonance imaging; T1WI: T1-weighted imaging; T2WI: T2-weighted imaging.

## Competing interests

The authors declare that they have no competing interests.

## Authors’ contributions

XL contributed mainly to the literature review and the writing of the manuscript. ZY revised the initial manuscript. Both authors read and approved the final manuscript.
